# Plasmacytoid dendritic cells downregulate the allergen induced T cell proliferation in a human *in vitro* allergy model

**DOI:** 10.1186/1476-9255-10-S1-P17

**Published:** 2013-08-14

**Authors:** Frank Schaumann, Armin Braun, Meike Müller

**Affiliations:** 1 Fraunhofer Institute for Toxicology and Experimental Medicine, Hannover, Germany; 2 Hannover Medical School, Hannover, Germany

## Background

The description of complex regulatory immunologic pathways represents a major difficulty during preclinical testing. Animal models are widely used for this purpose. In the study described herein, a human *in vitro* allergy model was established providing an alternative method to investigate allergy driven immune responses. The model should be able to show the influence of cellular components as i.e. plasmacytoid dendritic cells (pDC) or drug candidates. This might allow the avoidance of animal experiments and relates more closely to the human situation. For example, plasmacytoid dendritic cells (pDC) representing a DC subpopulation with major immunologic regulatory functions, were investigated to show a clear analogy to the in vivo situation in the model.

## Material and methods

Antigen-presenting cells were generated from CD14-positive monocytes obtained from whole blood of healthy or allergic donors. On day 5, APC were stimulated with house dust mite extract (HDM). On day 7, cells were harvested and DC were co-cultured with autologous CD4+ lymphocytes (ratio 1:10) in the presence of allergen with or without pDC or a immunomodulatory compound. Alternatively, PBMC were isolated from whole blood of healthy donors and stimulated with HDM ± pDC. The effects of pDC on the allergen-induced T cell proliferation were analyzed by ³H-thymidine incorporation. Cytokine secretion was measured in cell culture supernatants.

## Results

The generated and HDM-pulsed APC induced strong proliferation of the cocultured CD4+ lymphocytes compared to the unpulsed APC. The presence of pDC in the cocultures inhibited this proliferative response by about 50%. Likewise, the proliferation of HDM-stimulated PBMC was dose-dependently reduced by pDC, with the highest pDC dose nearly completely abolishing the allergen induced proliferation showing a significant deregulating functionality.

## Conclusions

An allergen-specific T cell immune response can reproducibly be performed using the human *in vitro* allergy model. The presence of pDC in this model clearly reduced the allergen-induced immune response, demonstrating the regulatory properties of this DC subpopulation depending on the stimulatory APC to pDC cell ratio. Hence, this model enables to mimic allergen-specific immune responses and furthermore regulatory mechanisms. In the future, this model thus might be applicable for efficacy testing of potential immunoregulatory cellular therapeutics or biomolecules with the advantages of avoiding animal experiments and enabling greater predictivity for the human situation.

